# The Importance of Uncertainty Analysis and Traceable Measurements in Routine Quantitative ^90^Y-PET Molecular Radiotherapy: A Multicenter Experience

**DOI:** 10.3390/ph16081142

**Published:** 2023-08-11

**Authors:** Marco D’Arienzo, Emilio Mezzenga, Amedeo Capotosti, Oreste Bagni, Luca Filippi, Marco Capogni, Luca Indovina, Anna Sarnelli

**Affiliations:** 1 Medical Physics Section, ASL Roma 6, Borgo Garibaldi 12, 00041 Rome, Italy; marco.darienzo@aslroma6.it; 2 UniCamillus International Medical University, 00131 Rome, Italy; 3 Medical Physics Unit, IRCCS Istituto Romagnolo per lo Studio dei Tumori (IRST) “Dino Amadori”, 47014 Meldola, Italy; emilio.mezzenga@irst.emr.it (E.M.); anna.sarnelli@irst.emr.it (A.S.); 4 Fondazione Policlinico Universitario A. Gemelli IRCCS, 00168 Roma, Italy; luca.indovina@policlinicogemelli.it; 5 Nuclear Medicine Department, Santa Maria Goretti Hospital, 04100 Latina, Italy; obagni1@gmail.com (O.B.); l.filippi@ausl.latina.it (L.F.); 6 ENEA, Italian National Institute of Ionizing Radiation Metrology, Via Anguillarese 301, 00123 Rome, Italy; marco.capogni@enea.it

**Keywords:** ^90^Y, PET, dosimetry, radionuclide therapy, quantitative accuracy, uncertainty analysis, MRT, scanner, multicenter

## Abstract

Molecular Radiation Therapy (MRT) is a valid therapeutic option for a wide range of malignancies, such as neuroendocrine tumors and liver cancers. In its practice, it is generally acknowledged that there is a need to evaluate the influence of different factors affecting the accuracy of dose estimates and to define the actions necessary to maintain treatment uncertainties at acceptable levels. The present study addresses the problem of uncertainty propagation in ^90^Y-PET quantification. We assessed the quantitative accuracy in reference conditions of three PET scanners (namely, Siemens Biograph mCT, Siemens Biograph mCT flow, and GE Discovery DST) available at three different Italian Nuclear Medicine centers. Specific aspects of uncertainty within the quantification chain have been addressed, including the uncertainty in the calibration procedure. A framework based on the Guide to the Expression of Uncertainty in Measurement (GUM) approach is proposed for modeling the uncertainty in the quantification processes, and ultimately, an estimation of the uncertainty achievable in clinical conditions is reported.

## 1. Introduction

Over the last two decades, there has been a massive increase in the development and use of radiopharmaceuticals for treating cancer, and the number of Molecular Radiation Therapy (MRT) treatments worldwide is soaring at an unprecedented rate [1]. Despite growing awareness of the expansion rate of MRT practice, it is generally recognized that quantitative imaging in MRT suffers from considerable inaccuracy and that dosimetry is significantly affected by uncertainties at every step of the dosimetric workflow [2,3,4,5]. As a consequence, when compared with conventional external beam radiotherapy, in which there are internationally agreed requirements for dose accuracy (<3% of a reference value), dosimetry in MRT still needs collaborative efforts to bring dosimetry practice to an acceptable standard.

In past years, two major international collaborative EURopean Association on national METrology institutes (EURAMET) projects have addressed the issues of traceability, accuracy, and uncertainties in MRT practice, developing some innovative solutions and proposing new approaches to the problem. The Metrology for Molecular Radiation Therapy (MetroMRT) project [6], concluded in 2015, aimed to develop the background metrology to support routine individual MRT patient dosimetry. The project identified major sources of error in the metrological processes involved in the evaluation of the absorbed dose and assessed uncertainty budgets in the dosimetric workflow. The following Metrology for Clinical Implementation of Dosimetry in Molecular Radiotherapy (MRTDosimetry) project [7] built on the results and outputs from the preceding MetroMRT project and ran for three years, finishing on 31 May 2019. These pan-European initiatives brought together expertise in metrology and nuclear medicine research to address the problem of the clinical implementation of dosimetry in molecular radiotherapy. With this in mind, both projects assessed the major processes and variables within the dose calculation procedure, evaluating their potential effect on the output result.

Quantitative ^90^Y-PET imaging has received much attention in the past decade [8,9,10,11,12], and the assessment of uncertainties in relation to the dose measurement chain (i.e., from a primary standard to a dosimetry calculation platform) has become a central issue for the evaluation of the efficacy and toxicity of Transarterial Radioembolization (TARE) [13,14,15,16,17]. Of note, one of the specific objectives of the MRTDosimetry project was to assess the internal pair production branching ratio and emission probabilities of ^90^Y, with the aim to enable improved quantitative imaging accuracy and dose estimation. The reason is that an accurate determination of the branching ratio for pair production is essential for accurate quantification and dosimetry.

Furthermore, recent studies carried out in the context of the above-mentioned EURAMET projects have addressed the issue of assessing an accurate uncertainty propagation schema in the quantification process [3] and in the dosimetry workflow [2]. D’Arienzo and Cox [3] performed uncertainty analysis in the calibration of an emission tomography system for quantitative imaging. In their study, using the general formula given in the Guide to the Expression of Uncertainty in Measurement (GUM) [18,19] for aggregating uncertainty components, the authors derived a practical relation to assess the combined standard uncertainty for the calibration factor of an emission tomography system. In another study, Gears and colleagues [2] proposed a comprehensive and accurate uncertainty propagation schema to evaluate the standard uncertainty in absorbed dose to a target. The paper has been published as an EANM guideline on uncertainty analysis for MRT absorbed dose calculations.

The aim of the present study is twofold. Firstly, it attempts to identify and describe a traceable validation procedure for ^90^Y-PET quantitative imaging in reference conditions. Secondly, the present research focuses on the problem of uncertainty propagation in the quantification workflow. As uncertainties propagate along each step of the quantification process, establishing a reliable scanner calibration procedure is essential to accurate activity quantification. With this in mind, we assessed the quantitative accuracy in reference conditions (cylindrical uniform geometry) of three PET scanners available at three different Italian Nuclear Medicine centers (namely, TOF Siemens Biograph mCT, TOF Siemens Biograph mCT flow, and GE Discovery DST). Specific aspects of uncertainty within the quantification chain have been addressed, including the uncertainty in the calibration procedure.

In the present paper, the three centers are referred to as indicated in Table 1. The workflow was organized as follows:

1. Three PET scanners available at three Italian centers were calibrated with the aim to recover the ^90^Y activity from ^90^Y-PET images. For all the PET scanners, the calibration procedure was performed using a water phantom uniformly filled with a known concentration of ^18^F-FDG to correlate the count rate to the phantom activity (Section 2.1).
2. After the calibration, for each scanner, a uniform cylindrical phantom containing ^90^Y was prepared with the aim to assess the quantitative accuracy of the scanner in reference conditions. Each uniform phantom was prepared following a traceable calibration methodology (Section 2.2). For the first two centers (GH, IRST), accurate activity concentration measurements of a stock ^90^Y radionuclidic solution were performed directly at the hospital using the ENEA-INMRI portable Triple-to-Double-Coincidence Ratio (TDCR). For one center (SMG), the activity concentration of the stock solution was measured using the on-site dose calibrator, traceable to a primary standard (Section 2.3).
3. Finally, the ability of each scanner to recover the activity concentration on the uniform phantom was assessed taking into account all possible correction factors (Section 2.4) and sources of uncertainty in the quantification processes (Section 2.5). The two TOF PET scanners available at the GH and IRST sites directly supported ^90^Y as a viable PET radionuclide, while ^90^Y was not present in the list of radionuclides accepted by the PET scanner available at the SMG center.
4. Ultimately, a framework is proposed for modeling the uncertainty in the quantification processes, along with an estimation of the uncertainty achievable in clinical conditions (Section 4).

In this study, the quantitative accuracy of ^90^Y-PET/CT was assessed on the following scanners (Table 1):

- Siemens Biograph mCT Flow: TOF PET/CT scanner (Siemens Medical Solutions, USA) available at IRCCS—Istituto Scientifico Romagnolo per lo Studio dei Tumori (IRST) “Dino Amadori” (Meldola, Italy);
- Siemens Biograph mCT: TOF PET/CT scanner (Siemens Medical Solutions USA) available at Fondazione Policlinino Universitario Agostino Gemelli IRCCS (Rome, Italy)
- GE Discovery DST BGO scanner (General Electric, Milwaukee, WI, USA) available at Ospedale Santa Maria Goretti (Latina, Italy)

## 2. Materials and Methods

### 2.1. Absolute Scanner Calibration

Absolute activity calibration factors are required to convert voxel values into a measure of absolute activity per voxel. A standard source configuration is generally recommended consisting of a phantom containing a known homogeneous activity concentration. The latter can be measured with the on-site dose calibrator. Traceability to the national standards laboratory for the geometry being measured is essential for activity determination and for uncertainty reduction. However, if activity is determined by a national laboratory, the final uncertainty can be reduced significantly.

Generally, all manufacturers have a standard procedure for the acquisition of radioactivity concentration calibration data, and PET absolute activity calibration is referred to in different terms by different manufacturers (e.g., well-counter calibration, radioactivity calibration factors, or SUV calibration). All PET scanners reported in Table 1 were calibrated using a traceable cylindrical phantom filled with a known amount of ^18^F (10 min-long scan for each calibration procedure).

The decay-corrected scanner calibration factor, *f*, can be defined as in Equation (1) [20]:

(1) $$f=\frac{R_{c}\left[\mathrm{counts}\right]}{A_{c}\left[\frac{\mathrm{kBq}}{\mathrm{mL}}\right]}$$

with $R_{c}$ representing the total counts inside a given Volume Of Interest (VOI) of the calibration phantom and $A_{c}$ the decay-corrected activity concentration in the calibration phantom, given by [21]:

(2) $$A_{c}=\frac{A_{0}}{V_{ph}}\exp\left(\frac{T_{\mathrm{cal}}-T_{0}}{T_{1/2}}\ln2\right)\left(\frac{T_{1/2}}{\ln2}\right)\left[1-\exp\left(-\frac{T_{\mathrm{acq}}}{T_{1/2}}\ln2\right)\right]$$

where

$A_{0}$ is the radionuclide activity used in the calibration procedure,
$V_{ph}$ is the volume of the phantom used in the calibration procedure,
$T_{0}$ is the acquisition start time,
$T_{\mathrm{cal}}$ is the reference calibration time,
$T_{1/2}$ is the radionuclide physical half-life, and
$T_{\mathrm{acq}}$ is the acquisition duration.

Equation (2) shows that accurate and precise activity measurements of the quantity $A_{0}$ are an essential pre-requisite of quantitative imaging and dosimetry. The ^18^F activity ($A_{0}$ in Equation (2)) was measured using on-site dose calibrators, traceable to primary standards. Activity concentration measurements were performed with an accuracy within 1.7% (at k = 1 level) for the GH and IRST center and 2% (at k = 1 level) for the SMG center (Table 1).

### 2.2. Preparation of a Traceable Phantom for ^90^Y-PET Studies

Quantitative imaging studies rely on phantoms containing a traceable amount of activity concentration. As a general rule, the preparation of a calibrated phantom may be prone to a number of uncertainties. However, the preparation of reference phantoms with a metrological approach provides traceability to measurement results.

In the present study, Diethylenetriaminepentaacetic Acid (DTPA) at a concentration of about 50 μg/g was used to prevent radioactive ^90^Y from sticking to the phantom walls and to guarantee a homogeneous radionuclide solution. A cylindrical uniform phantom (without any insert) was pre-filled with this carrier solution 12 h prior to the addition of ^90^YCl_3_, thereby contributing to sealing the phantom’s inner walls and reducing sticking or plating activity.

The knowledge of the ^90^Y activity concentration is required to assess the calibration factor (*f*) through Equation (1). Therefore, accurate volume measurements were required for accurate activity concentration estimates. The volume *V* of a liquid solution can be conveniently assessed from mass measurements using a calibrated balance and then introducing the liquid density ρ as follows:

(3) $$V=\frac{m}{\rho }$$

where *m* is the mass of the radionuclide solution. In the present study, we assumed ρ = 1 g/cm^3^. In order to minimize weighing uncertainties, small masses were measured using a digital four-decimal place balance provided with a draft shield to prevent air turbulence. Phantom volumes were assessed by the difference, weighing the phantom prior to and after its filling.

As a general rule, the significant factors that contribute to measurement uncertainty across the weighing range are repeatability, eccentricity (the error associated with not placing the weight in the center of the weighing pan), nonlinearity (the error due to the nonlinear behavior of the balance upon increasing the load on the weighing pan), and sensitivity (i.e., systematic deviation). If analytic balances are used for the measurements of small masses, uncertainties below 0.001% can be achieved.

### 2.3. ^90^Y Activity Concentration Measurements

For the two centers (GH and IRST) using the Siemens Biograph mCT and Siemens Biograph mCT Flow system, accurate activity concentration measurements of the ^90^Y radionuclidic solution were performed on-site using the ENEA-INMRI portable TDCR. The TDCR method is a primary absolute activity measurement technique specially developed for pure beta- and pure EC-emitters’ activity determination [22]. The activity concentration of the stock ^90^Y solution was determined with an uncertainty of ±1% (at k = 1 level). For the center SMG, the activity concentration of the stock ^90^Y solution was measured using the on-site dose calibrator, traceable to a primary standard. In this case, activity concentration measurements were performed with an accuracy within ±2.5% (at k = 1 level).

### 2.4. Quantitative Imaging on ^90^Y Clinical Acquisitions

In order to validate the calibration procedure, the uniform ^90^Y cylindrical phantom (see Section 2.2 for details on the preparation) was imaged, and PET/CT phantom images acquired by each center were reconstructed as reported in Table 2. Each dataset was analyzed using the PMOD software (Version 3.9, PMOD Technologies Ltd., Switzerland). A cylindrical VOI was coaxially outlined at the center of the phantom. To minimize edge effects, the cylindrical VOI was selected excluding the inner boundaries of the phantom (3 cm distance from the edges).

Considering that quantification for different positron-emitting radionuclides by PET systems can be performed with a simple rescaling of pixel values based on (i) the half-life and (ii) the branching ratio for positron emission of the investigated/injected radionuclide, the counts within the VOI need to be corrected as described below.

#### 2.4.1. Half-Life Correction

In the case of ^90^Y clinical imaging, an adjusted decay constant must be introduced in the system in order to account for the different half-lives of the radionuclide used in the calibration procedure and that of ^90^Y. This correction is generally performed by the PET scanner. If the scanner does not support this option, a Decay Correction Factor (DCF) must be applied to the reconstructed data using the surrogate radionuclide *X* reported in Equation (4) [23]:

(4) $$DCF\left(X\to^{90}Y\right)=\frac{T_{1/2}\left(X\right)}{T_{1/2}{(}^{90}Y)}\cdot \frac{1-exp\left[-ln\left(2\right)\cdot \frac{T_{acq}}{T_{1/2}\left(X\right)}\right]}{1-exp\left[-ln\left(2\right)\cdot \frac{T_{acq}}{T_{1/2}{(}^{90}Y)}\right]}$$

$T_{1/2}\left(X\right)$ being the physical half-life of the radionuclide *X* and $T_{acq}$ the PET acquisition duration.

#### 2.4.2. Branching Ratio Correction

In addition, the number of counts needs to be rescaled by the ratio of the β^+^ emission probability of the surrogate radionuclide and that of ^90^Y. In order to obtain the ^90^Y activity concentration in terms of kBq/mL, the total number of counts in the selected VOI (*R*) needs to be ultimately rescaled by the ratio of the β^+^ emission probability of the used radionuclide ($w_{\beta^{+}}^{X}$) and that of ^90^Y ($w_{\beta^{+}}^{90Y}$) as:

(5) $$R_{{}^{90}Y}=\frac{R\cdot (w_{\beta^{+}}^{X})}{\left(w_{\beta^{+}}^{{}^{90}Y}\right)}\left[\mathrm{counts}\right]$$

where $R_{90Y}$ is the number of counts of ^90^Y assessed on the VOI. For scanners that do not support ^90^Y as a viable radionuclide option, a number of surrogate radionuclides have been used in the published literature. The GE Discovery DST scanner used in the present study does not provide an option for specifying imaging-related parameters for the ^90^Y isotope, while both Siemens Biograph mCT scanners support ^90^Y as a viable radionuclide option (i.e., ^90^Y is available from the scanner console’s radionuclide list).

#### 2.4.3. ^90^Y Quantification

Once the PET scanner has been properly calibrated and ^90^Y images have been acquired, the absolute ^90^Y activity concentration in any clinical setting, $A_{c}^{clin}$, can be assessed combining Equations (1), (2), and (5):

(6) $$A_{c}^{clin}=\frac{R_{{}^{90}Y}}{f}=\frac{R}{R_{c}}\frac{\left(w_{\beta^{+}}^{X}\right)}{\left(w_{\beta^{+}}^{{}^{90}Y}\right)}\cdot \frac{A_{0}}{V_{ph}}\exp\left(\frac{T_{\mathrm{cal}}-T_{0}}{T_{1/2}}\ln2\right)\left(\frac{T_{1/2}}{\ln2}\right)\left[1-\exp\left(-\frac{T_{\mathrm{acq}}}{T_{1/2}}\ln2\right)\right]$$

### 2.5. Evaluation of Uncertainty

The Guide to the Expression of Uncertainty in Measurement (GUM) [19] is the standard for the evaluation of measurement uncertainty in metrology. Let $Q_{1},Q_{2},\ldots ,Q_{n}$ denote a set of *n* “input” quantities and *Y* an “output” quantity or measurand. The GUM considers the generic measurement model:

$$Y=f(Q_{1},Q_{2},\ldots ,Q_{n}),$$

that is a known functional relationship between the input and the output quantities. Given estimates $q_{1},q_{2},\ldots ,q_{n}$ of the input quantities, the GUM uses

$$y=f(q_{1},q_{2},\ldots ,q_{n})$$

as the corresponding estimate of *Y*. Further, given standard uncertainties $u\left(q_{1}\right),u\left(q_{2}\right),\ldots ,$$u(q_{n})$ associated with $q_{1},q_{2},\ldots ,q_{n}$, the GUM applies the Law of Propagation of Uncertainty (LPU) to evaluate the combined standard uncertainty u(y) associated with *y*. For independent input quantities, the LPU is described by the following expression:

(7) $$u^{2}\left(y\right)={\left(\frac{\partial f}{\partial q_{1}}\right)}^{2}\;u^{2}\left(q_{1}\right)+{\left(\frac{\partial f}{\partial q_{2}}\right)}^{2}u^{2}\left(q_{2}\right)+\cdots +{\left(\frac{\partial f}{\partial q_{n}}\right)}^{2}u^{2}\left(q_{n}\right),$$

in which $\partial f/\partial q_{i}$ denotes $\partial f/\partial Q_{i}$ evaluated at $q_{1},q_{2},\ldots ,q_{n}$.

Equation (7) was used in the present study to assess the relative uncertainty in the activity concentration, $u(A_{c}^{clin})$, as determined by Equation (6).

## 3. Results

Equation (7) gives the general form for the relative standard uncertainty associated with *y*. By applying this relation to Equation (6), the combined standard uncertainty in the final activity concentration, $u(A_{c}^{clin})$, can be obtained. D’Arienzo and Cox [3] have demonstrated that if the acquisition time is much smaller than the radionuclide half-life (i.e., $T_{\mathrm{acq}}\ll T_{1/2}$, as it is for ^90^Y), in terms of relative standard uncertainties, the uncertainty in the calibration factor, *f*, reduces to:

(8) $$\begin{array}{cc} u_{\mathrm{rel}}^{2}\left(f\right)\; & \approx u_{\mathrm{rel}}^{2}\left(R\right)+u_{\mathrm{rel}}^{2}\left(V_{p_{h}}\right)+u_{\mathrm{rel}}^{2}\left(A_{0}\right)\\ \; & +{\left[\frac{(T_{0}-T_{\mathrm{cal}})\ln2}{T_{1/2}}\right]}^{2}\left[u_{\mathrm{rel}}^{2}\left(T_{0}-T_{\mathrm{cal}}\right)+u_{\mathrm{rel}}^{2}\left(T_{1/2}\right)\right]+u_{\mathrm{rel}}^{2}\left(T_{\mathrm{acq}}\right). \end{array}$$

where $u_{rel}\left(R\right)$ is the relative uncertainty in the detected counts, $u_{rel}\left(V_{ph}\right)$ the relative standard uncertainty associated with the volume measurement (which typically translates into weighing of masses) and $u_{rel}\left(A_{0}\right)$ the relative uncertainty in the calibration activity. The quantity $u_{rel}\left(T_{0}-T_{\mathrm{cal}}\right)$ in Equation (8) is the relative standard uncertainty associated with the time difference between the acquisition start time $T_{0}$ and the reference calibration time $T_{\mathrm{cal}}$. The relative time offset between the two clocks used to determine $T_{0}$ and $T_{\mathrm{cal}}$ can be considered representative of $u_{rel}\left(T_{0}-T_{\mathrm{cal}}\right)$. Ultimately, $u_{\mathrm{rel}}\left(T_{1/2}\right)$ and $u_{\mathrm{rel}}\left(T_{\mathrm{acq}}\right)$ represent the uncertainty in the radionuclide half-life and the acquisition time, respectively.

The final combined relative uncertainty in the activity concentration, $u_{\mathrm{rel}}\left(A_{c}^{clin}\right)$, can be obtained by adding in quadrature the relative uncertainties of the branching ratios $u_{\mathrm{rel}}\left(w_{\beta^{+}}^{X}\right)$, $u_{\mathrm{rel}}\left(w_{\beta^{+}}^{90Y}\right)$ and the relative uncertainty on the total detected ^90^Y counts, $u_{rel}\left(R_{c}\right)$, as:

(9) $$\begin{array}{cc} u_{\mathrm{rel}}^{2}\left(A_{c}^{clin}\right)\; & \approx u_{\mathrm{rel}}^{2}\left(R\right)+u_{\mathrm{rel}}^{2}\left(V_{p_{h}}\right)+u_{\mathrm{rel}}^{2}\left(A_{0}\right)\\ \; & +{\left[\frac{(T_{0}-T_{\mathrm{cal}})\ln2}{T_{1/2}}\right]}^{2}\left[u_{\mathrm{rel}}^{2}\left(T_{0}-T_{\mathrm{cal}}\right)+u_{\mathrm{rel}}^{2}\left(T_{1/2}\right)\right]+u_{\mathrm{rel}}^{2}\left(T_{\mathrm{acq}}\right)\\ \; & +u_{\mathrm{rel}}^{2}\left(w_{\beta^{+}}^{X}\right)+u_{\mathrm{rel}}^{2}\left(w_{\beta^{+}}^{{}^{90}Y}\right)+u_{rel}^{2}\left(R_{c}\right). \end{array}$$

**Figure 1:**
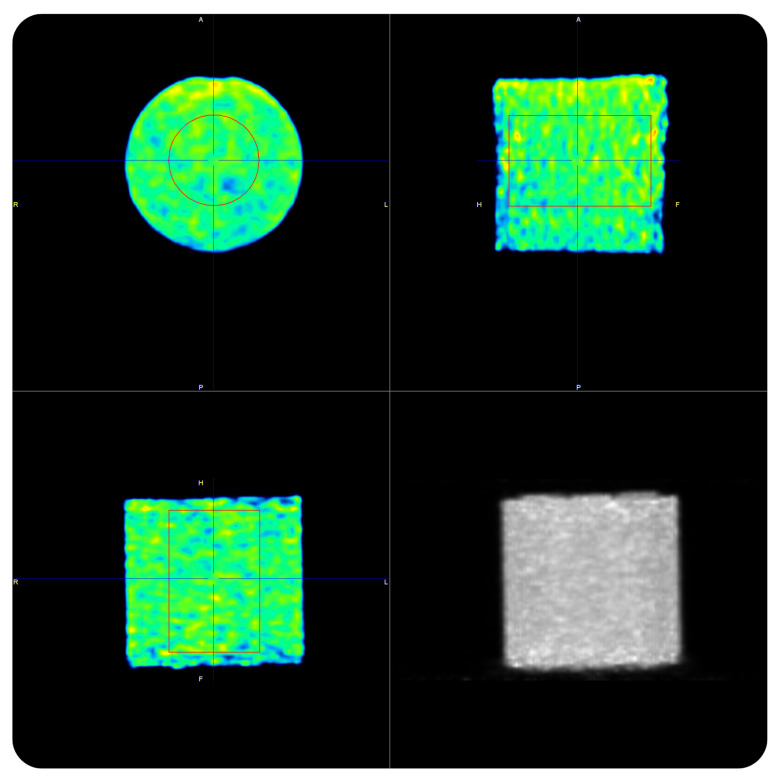
^90^Y-PET acquisition of the uniform phantom on the Siemens Biograph mCT Flow.

Following the above-mentioned procedure, we validated the vendor calibration procedure assessing the ability of each PET scanner to accurately recover the ^90^Y activity concentration in the uniform ^90^Y phantom. Overnight PET acquisitions of the uniform phantoms (Figure 1) were performed.

Table 3 compares the reconstructed ^90^Y activity concentrations versus the measured values for each center, while Table 4 reports the relative uncertainties computed for each center, together with the relative activity uncertainty based on Equation (9).

The uncertainty in the number of counts, u(R), can be determined with different approaches, depending on the counting statistics. Assuming that the process is dominated by Poisson distributed noise, the uncertainty on the total detected counts can be considered equal to the square root of the total number of detected counts (i.e., $u\left(R\right)=\sqrt{R}$). As a general rule, ^18^F-PET imaging is well described by a Poisson-like distribution. Therefore, in this study, the relative uncertainty in the total counts inside a given VOI of the calibration phantom was determined as $u_{rel}\left(R_{c}\right)=\sqrt{R_{c}}/R_{c}$. In particular, $u_{rel}\left(R_{c}\right)$ was conservatively estimated to be about 0.5% for all centers. In fact, the calibration procedure is generally performed using ^18^F and collecting at least $10^{6}$ counts in the VOI. However, this approach should be used with caution for low counting statistics, as in the case of ^90^Y-PET, where extremely low count rates are generally observed especially during clinical acquisitions. In the present study, the uncertainty in the ^90^Y reconstructed images, $u_{\mathrm{rel}}\left(R\right)$, was determined in terms of the Coefficient Of Variation (COV) (i.e., the ratio of the standard deviation (sd) to the mean value, i.e., $u_{rel}\left(R\right)=sd/R$). The uncertainty in the volume of the phantom used in the calibration procedure, $u_{\mathrm{rel}}\left(V_{ph}\right)$, was estimated to be about 0.5%. The uncertainty in the calibration activity, $u_{\mathrm{rel}}\left(A_{0}\right)$, was determined to be 2.0% (k = 1) for the SMG center and 1.7% (k = 1) for IRST and GH. All participating centers measured the calibration activity using the on-site radionuclide dose calibrator traceable to national laboratories. Methods to determine dose calibrator uncertainty are extensively described by Gadd et al. [24]. With a conservative approach, the uncertainty in $u_{\mathrm{rel}}\left(T_{cal}\right)$, $u_{\mathrm{rel}}\left(T_{0}\right)$, $u_{\mathrm{rel}}\left(T_{acq}\right)$, and $u_{\mathrm{rel}}\left(T_{1/2}\right)$ was assumed to be in the order of 0.1%. Ultimately, the uncertainty in the decay branching ratio of $u_{\mathrm{rel}}\left(w_{\beta^{+}}^{90Y}\right)$ and $u_{\mathrm{rel}}\left(w_{\beta^{+}}^{X}\right)$ was assumed to be 1.2% [25] and 0.2% [26], respectively.

**Figure 2:**
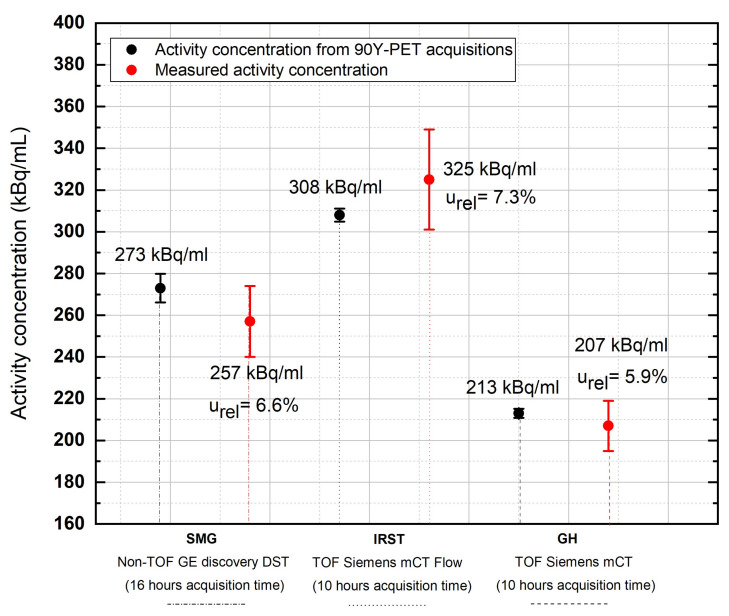
Comparison of recovered activity concentration values in the uniform phantom versus true activity concentration values at the three Italian sites. Acquisition time: 16 h for SMG; 10 h for IRST and GH.

The relative difference in the reconstructed activity concentration varied from −5.9% (SMG) to +5.5% (IRST). Of note, the two centers (GH and IRST) operating with the same PET scanner used different post-reconstruction Gaussian filter sizes (i.e., 6 mm for GH and 2 mm for IRST). Most likely, the lower uncertainty in the counts associated with the GH center ($u_{R}=5.5\%$) can be attributed to the use of a larger Gaussian filter, responsible for a greater smoothing of the image. This ultimately resulted in an overall lower uncertainty on the recovered activity, $u_{rel}\left(A_{c}^{clin}\right)$ (5.9% vs. 7.3%; Figure 2 and Table 4).

**Figure 3:**
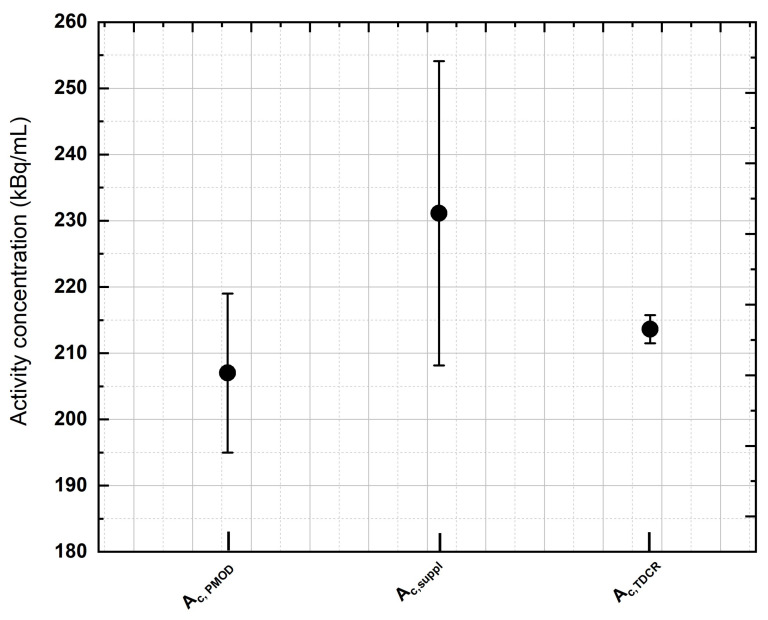
An example of activity concentration assessment performed during the study. The activity concentration recovered with the PMOD 3.9 software ($A_{c,PMOD}$) in the uniform phantom imaged at GH was compared with the activity concentration provided by the supplier ($A_{c,suppl}$) (±5% uncertainty, coverage factor of k = 1) and the activity concentration measured with the ENEA-INMRI portable TDCR portable system ($A_{c,TDCR}$) (±1.0%, coverage factor of k = 1). All activity concentration values lie within the stated uncertainties, with the latter method (TDCR) providing the most-accurate measurement.

**Figure 4:**
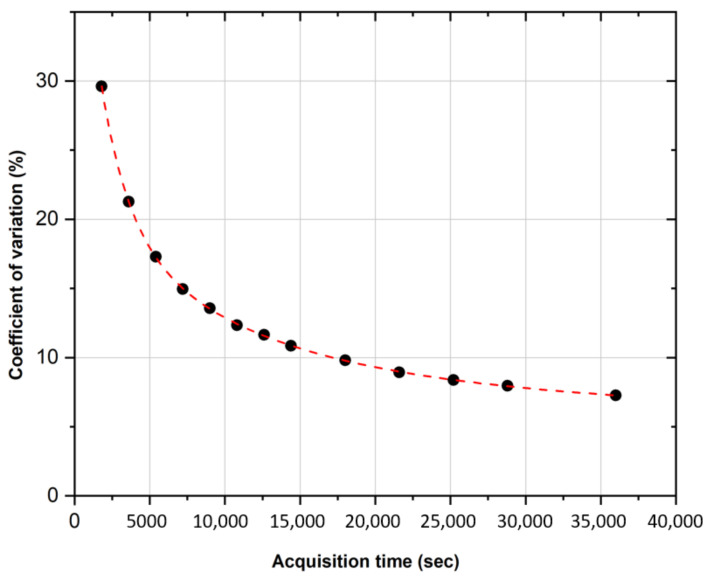
COV evaluation of the activity concentration versus PET acquisition time. Acquisition on the cylindrical phantom uniformly filled with ^90^Y.

Figure 3 compares the activity concentration recovered with the PMOD 3.9 software ($A_{c,PMOD}$) in the uniform phantom imaged at the GH center with the activity concentration provided by the supplier (±5% uncertainty at the k = 1 level ($A_{c,suppl}$)) and the activity concentration measured with the ENEA-INMRI portable TDCR system (±1.0% at the k = 1 level ($A_{c,TDCR}$)). For the same center, Figure 4 shows the relative standard deviation in the recovered activity concentration as a function of the acquisition time. A total of thirteen acquisitions were performed: from 30 min to 4 h (increasing each new acquisition by 30 min) and from 4 h to 10 h (increasing each new acquisition by 1 h). Of note, for typical clinical acquisitions (±30 min), the COV is in the order of 30% due to the extremely low counts and high random fraction associated with ^90^Y-$\beta^{+}$ decay. This may possibly introduce a relevant source of uncertainty in patient dosimetry.

## 4. Discussion

The determination of the absorbed dose in MRT practice is an essential part of the management of the treatment of each individual patient. In fact, it is a requirement in EC Directive 97/43 Euratom, which states that, for radiotherapeutic purposes, *"exposures of target volumes shall be individually planned"*. The purpose of this study is to establish a traceable workflow for accurate quantitative ^90^Y-PET imaging with the intention of relating the uncertainty of the output quantity (recovered activity concentration) to the uncertainty of the input data. In fact, in clinical practice, quantitative data are used for radiation dose assessment; therefore, uncertainties in the initial quantities propagate directly into the dose calculation.

The issue of the role and involvement of metrology institutes in quantitative imaging and the entire dosimetric process is not new and has been addressed by several authors in past [4,6,7] and recent [27] research. In conventional External Beam Radiotherapy (EBRT), individual patient dosimetry is mandatory, strictly controlled according to agreed protocols, and there is full traceability to primary standards. In contrast, for nuclear medicine, the role of the metrology institutes is less clear and the calculation of the administered absorbed doses is not traceable in the same manner as EBRT [27]. The need for metrology support is particularly true for difficult-to-measure radionuclides such as ^90^Y [27].

In the present study, the low counting statistics related to ^90^Y-PET acquisitions ($u_{\mathrm{rel}}\left(R\right)$), the uncertainty of the source activity used in the PET system calibration ($u_{\mathrm{rel}}\left(A_{0}\right)$), and the uncertainty in the ^90^Y internal pair production branching ratio ($u_{\mathrm{rel}}\left(w_{\beta^{+}}^{90Y}\right)$) are the main factors contributing to the final uncertainty of the recovered activity concentration.

The issue of poor image quality related to the low counting statistics associated with the ^90^Y internal pair production has already been addressed in several literature works and will not be covered here. The reader is referred to [28,29,30,31] for further insights

Past research [32] showed the measurement of the calibration factor as being one of the major sources of uncertainties in the dose measurements (together with the uncertainty related to the positive bias due to the intrinsic radioactivity of scanner’s crystals). In this work, thanks to the long acquisition time, the relative uncertainties in the recovered ^90^Y activity concentration were found to be in the range of ≃6–7% depending on the scanner model and, most importantly, on the availability of the TOF technology. It should be noted that, in the present study, the overnight phantom acquisition reflected a relatively uniform image, thereby providing a coefficient of variation in the counting statistics ($u_{\mathrm{rel}}\left(R\right)$) (Equation (9)) in the order of 5.5–7%. In clinical conditions, a shorter acquisition time is likely to produce larger uncertainties, which may impair both qualitative and quantitative results.

Most notably, few researchers have addressed the importance of phantom preparation. In a past study, Sunderland and colleagues [33] demonstrated that technical error in phantom filling is one of the primary reasons for the exclusion of PET/CT scanners from clinical trials. In addition, the adsorption of radionuclides on the inner walls of plastic phantoms may lead to an inhomogeneous radionuclide distribution, which can negatively affect quantitative imaging studies [34]. Therefore, the preparation of a carrier solution is recommended. The use of tap water should be avoided as minerals and other chemical impurities might stick to the phantom walls or combine with the radiopharmaceuticals, changing the radionuclide distribution. For ^90^Y-PET studies, ^90^YCl_3_ in an aqueous solution of 0.1 mol/dm3 hydrochloric acid also containing inactive Yttrium at a concentration of about 50 μg/g can be used as a carrier solution. Alternatively, Diethylenetriaminepentaacetic Acid (DTPA) or Ethylenediaminetetraacetic Acid (ETPA) at a concentration of about 50 μg/g can be used to prevent radioactive ^90^Y from sticking to the phantom walls and to guarantee a homogeneous radionuclide solution. It is recommended that all containers be pre-filled with the carrier 12 h prior to the addition of radioactive ^90^YCl_3_. This will help to “seal” the surface and reduce sticking or plating activity. All containers should be emptied, dried, and the used carrier discarded before activity is added. As a general rule, the preparation of a stock solution is recommended. Radioactive ^90^Y provided by the supplier should be diluted using the carrier solution to the desired volume and concentration. The activity concentration should be determined by measuring an aliquot of the stock solution in terms of the activity per unit mass (or volume). This can then be used to determine the activity of all subsequent sources produced from this stock solution. Filling of the phantoms should be performed using a calibrated (preferably four decimal places) analytic scientific scale and with routine double or triple weighting of the sources. The overall uncertainty in the activity concentration determined using this method is dependent on the precision of the scale being used, as well as the accuracy of the method used to determine the activity concentration of the solution. Radioactivity should be dispensed using calibrated pipette devices or syringes and ensuring that no air bubbles remain in the phantom. If large background volumes are used for calibration purposes, the phantom can be filled with non-radioactive water to measure the fillable volume (and to confirm the phantom is watertight with no leaks). When filling large phantom volumes, a funnel should be used. When the phantom is nearly full, the funnel can be removed and a syringe used to complete the filling process, thereby preventing spillage of radioactive water from the background compartment.

One of the major drawbacks of quantitative imaging with ^90^Y microspheres is related to the quick microsphere sedimentation over time. Therefore, in order to have a homogeneous solution, phantom calibration studies need to be performed with ^90^Y chloride (^90^YCl_3_) instead of ^90^Y microspheres. The instrument typically used to measure the administered activity to patients in nuclear medicine procedures is the radionuclide dose calibrator. Recent [35,36,37] and previous [38] findings reported difficulties of measuring ^90^Y chloride and other beta emitters using clinically available ionization chambers. This is because dose calibrators available in the clinical nuclear medicine contextperform activity measurements of beta-emitting radionuclides indirectly, by detecting bremsstrahlung emissions. Bremsstrahlung production is highly dependent on the source material, its container, and the calibrator chamber wall. The ionization current also depends on the probability of electron detection within the chamber, which varies with electron energy and individual dose calibrator construction. Moreover, slight variations in the container wall thickness, solution volume, or location within the well can lead to an increase in the overall assay uncertainty when using the manufacturer-supplied calibration factor, which is typically traceable to national standards. For activity measurements of ^90^YCl_3_ at a clinical level, it is expected that radionuclide dose calibrators provide accuracy within ±5% (at k = 2 level) [24,39]. However, if the activity is determined by a national metrology institute, uncertainty on the activity concentration can be reduced dramatically. Primary activity standards for ^90^Y are widely available, and measurement uncertainties below 1% can be achieved [40,41].

Ultimately, another key factor impacting the achievable quantitative accuracy is the uncertainty in the ^90^Y branching ratio. In 2007, Selwyn et al. [42] determined the branching ratio related to $\beta^{+}/\beta^{-}$ pair production during ^90^Y decay to be $\left(31.86\pm 0.47\right)\times 10^{-6}$, following de-excitation from the $0^{+}$ excited state of ^90^Zr. Recently, the internal pair production branching ratio of ^90^Y was experimentally determined by the Czech Metrology Institute (CMI) and the National Institute of Standards and Technology (NIST). Dryák and Šolc [25] provided a branching ratio of $\left(32.6\pm 0.4\right)\times 10^{-6}$. Along the same lines, Pibida and colleagues [43] estimated the internal pair production branching ratio to be $\left(32\pm 1.5\right)\times 10^{-6}$ (k = 1), resulting in being within one standard uncertainty with the recommended value of $\left(32.6\pm 0.7\right)\times 10^{-6}$ (k = 1) from the Decay Data Evaluation Project (DDEP) database [26].

Accurate determination of the absorbed dose from ^90^Y-PET requires accurate evaluation of the radiopharmaceutical localization, adding considerable additional complexity to the dosimetry workflow. The translation of ^90^Y-PET quantitative data into an accurate dose distribution within the patient is complex, and at present, there is no clear understanding or quantification of the uncertainty involved in ^90^Y-PET image-based dosimetry in clinical conditions. For clinical reasons, an overall uncertainty below 10% is desirable, and future research should be devoted to identifying major sources of error in the processes involved in the measurement of the absorbed dose and quantify them in terms of the modeling and uncertainty analysis.

## 5. Conclusions

In this study, we have proposed a workflow for ^90^Y-PET validation, along with a procedure to assess the uncertainty in the recovered activity, based on the law of propagation of uncertainties (GUM uncertainty approach). In this work, the relative standard uncertainty in the recovered activity was in the range ≃6–7%. However, the shorter acquisition time generally used during clinical acquisition is likely to produce larger uncertainties, which may impair both qualitative and quantitative results. More generally, the low counting statistics related to ^90^Y-PET acquisitions, the uncertainty of the source activity used in the PET system calibration, and the uncertainty in the ^90^Y internal pair production branching ratio appear to be the main factors contributing to the final uncertainty of the recovered activity concentration.

## Figures and Tables

**Table 1:** Italian centers participating in the study, along with their scanners and related calibration source.

Site	Scanner Model	^90^Y-Supported	PET Calibration Source
Santa Maria Goretti Hospital (SMG), Latina ${}^{†}$	GE Discovery DST (General Electric, Milwaukee, WI, USA)	No	Cylindrical phantom, ^18^F solution (2% uncertainty, k = 1)
Gemelli Hospital (GH), Rome ${}^{‡}$	TOF Siemens Biograph mCT (Siemens Medical Solutions USA)	Yes	Cylindrical phantom, ^18^F solution (1.7% uncertainty, k = 1)
IRST Tumor Center (IRST), Meldola ${}^{‡}$	TOF Siemens Biograph mCT Flow (Siemens Medical Solutions USA)	Yes	Cylindrical phantom, ^18^F solution (1.7% uncertainty, k = 1)

${}^{†}$ The GE Discovery DST scanner used in the present study does not provide an option for specifying imaging-related parameters for the ^90^Y radionuclide. ${}^{‡}$ Siemens Biograph mCT scanners support ^90^Y as a viable radionuclide option (i.e., ^90^Y is available from the scanner console’s radionuclide list).

**Table 2:** PET/CT image acquisition and reconstruction parameters used by the centers.

Site	True ^90^Y Activity	Reconstruction Algorithm	Applied Corrections	CT Scan Parameters
SMG—Latina (GE Discovery DST), 16 h scan	273 kBq/mL	3D OSEM (15 subsets, 2 iterations)	Uniformity, attenuation scatter, decay, dead-time, and randomness	120 kV, 60 mAs
GH—Rome (TOF Siemens Biograph mCT), 10 h scan	213 kBq/mL	3D TOF-OSEM (21 subsets, 1 iteration)	Uniformity, attenuation scatter, decay, dead-time, and randomness	120 kV, 50 mAs
IRST—Meldola (TOF Siemens Biograph mCT Flow), 10 h scan	308 kBq/mL	3D TOF-OSEM (21 subsets, 1 iteration)	Uniformity, attenuation scatter, decay, dead-time, and randomness	120 kV, 80 mAs

**Table 3:** ^90^Y-PET quantitative accuracy for each center. The ^90^Y true phantom activity concentration was measured with the on-site dose calibrator for the SMG hospital and with the portable ENEA TDCR for both the IRST and GH centers. Uncertainties in the true phantom activity are reported with a coverage factor of k = 1.

	GE Discovery DST (SMG)	Siemens Biograph mCT Flow (IRST)	Siemens Biograph mCT (GH)
True phantom $A_{c}$	(273 ± 7) kBq/mL	(308 ± 3) kBq/mL	(213 ± 2) kBq/mL
Recovered $A_{c}$	(257 ± 17) kBq/mL	(325 ± 24) kBq/mL	(207 ± 12) kBq/mL
Deviation	−5.9%	+5.5%	−2.8%

**Table 4:** ^90^Y-PET quantitative accuracy in the uniform cylindrical phantom. Relative uncertainties evaluated for each center and variables considered in this study.

Uncertainty Component	GE Discovery DST (SMG)	Siemens Biograph mCT Flow (IRST)	Siemens Biograph mCT (GH)
$u_{\mathrm{rel}}\left(R_{c}\right)$	0.5%	0.5%	0.5%
$u_{\mathrm{rel}}\left(R\right)$	6.2%	7.0%	5.5%
$u_{\mathrm{rel}}\left(V_{ph}\right)$	0.5%	0.5%	0.5%
$u_{\mathrm{rel}}\left(A_{0}\right)$	2.0%	1.7%	1.7%
$u_{\mathrm{rel}}\left(T_{0}\right)$	0.1%	0.1%	0.1%
$u_{\mathrm{rel}}\left(T_{cal}\right)$	0.1%	0.1%	0.1%
$u_{\mathrm{rel}}\left(T_{1/2}\right)$	0.1%	0.1%	0.1%
$u_{\mathrm{rel}}\left(T_{acq}\right)$	0.1%	0.1%	0.1%
$u_{\mathrm{rel}}\left(w_{\beta^{+}}^{90Y}\right)$	1.2%	1.2%	1.2%
$u_{\mathrm{rel}}\left(w_{\beta^{+}}^{X}\right)$	0.2%	0.2%	0.2%
Acquisition time	16 h	10 h	10 h
** $u_{rel}\left(A_{c}^{clin}\right)$ **	**6.6%**	**7.3%**	**5.9%**

## Data Availability

Not applicable.

## Abbreviations

The following abbreviations are used in this manuscript:

MRT	Molecular Radiation Therapy
MetroMRT	Metrology for Molecular Radiation Therapy
MRTDosimetry	Molecular Radiation Therapy Dosimetry
TARE	Transarterial Radioembolization
GUM	Guide to the Expression of Uncertainty in Measurements
EANM	European Association of Nuclear Medicine
TDCR	Triple-to-Double-Coincidence Ratio
DTPA	Diethylenetriaminepentaacetic Acid
PET	Positron Emission Tomography
TOF	Time Of Flight

## References

[1] Sgouros G., Bodei L., McDevitt M.R., Nedrow J.R. (2020). Radiopharmaceutical therapy in cancer: Clinical advances and challenges. Nat. Rev. Drug Discov..

[2] Gear J.I., Cox M.G., Gustafsson J., Gleisner K.S., Murray I., Glatting G., Konijnenberg M. (2018). EANM practical guidance on uncertainty analysis for molecular ra-diotherapy absorbed dose calculations. Eur. J. Nucl. Med. Mol. Imaging.

[3] D’Arienzo M., Cox M. (2017). Uncertainty analysis in the calibration of an emission tomography system for quantitative imaging. Comput. Math. Methods Med..

[4] D’Arienzo M., Capogni M., Smyth V., Cox M., Johansson L., Solc J., Bobin C., Rabus H., Joulaeizadeh L. (2014). Metrological Issues in Molecular Radiotherapy. EPJ Web Conf..

[5] Tran-Gia J., Salas-Ramirez M., Lassmann M. (2020). What you see is not what you get: On the accuracy of voxel-based dosimetry in molecular radiotherapy. J. Nucl. Med..

[6] MetroMRT—Metrology for Molecular Radiation Therapy. http://projects.npl.co.uk/metromrt/.

[7] MRTDosimetry. https://osf.io/69nge/.

[8] Pasciak A.S., Bourgeois A.C., McKinney J.M., Chang T.T., Osborne D.R., Acuff S.N., Bradley Y.C. (2014). Radioembolization and the dynamic role of 90Y-PET/CT. Front. Oncol..

[9] Ungania S., D’Arienzo M., Mezzenga E., Pizzi G., Vallati G., Ianiro A., Rea S., Sciuto R., Soriani A., Strigari L. (2022). A Workflow for Dosimetry of 90Y Radio-embolization Based on Quantitative 99mTc-MAA SPECT/CT Imaging and a 3D-Printed Phantom. Appl. Sci..

[10] Willowson K.P., Tapner M., Bailey D.L., QUEST Investigator Team (2015). A multicenter comparison of quantitative 90 Y-PET/CT for dosimetric purposes after radioembolization with resin microspheres: The QUEST phantom study. Eur. J. Nucl. Med. Mol. Imaging.

[11] Gates V.L., Esmail A.A., Marshall K., Spies S., Salem R. (2011). Internal pair production of 90Y permits hepatic localization of microspheres using routine PET: Proof of concept. J. Nucl. Med..

[12] D’Arienzo M. (2013). Emission of *β*+ Particles Via Internal Pair Production in the 0+ – 0+ Transition of 90Zr: Historical Background and Current Applications in Nuclear Medicine Imaging. Atoms.

[13] Spreafico C., Maccauro M., Mazzaferro V., Chiesa C. (2014). The dosimetric importance of the number of 90 Y microspheres in liver transarterial radioembolization (TARE). Eur. J. Nucl. Med. Mol. Imaging.

[14] Hardy-Abeloos C., Lazarev S., Ru M., Kim E., Fischman A., Moshier E., Rosenzweig K., Buckstein M. (2019). Safety and efficacy of liver stereotactic body radiation therapy for hepatocellular carcinoma after segmental transarterial radioembolization. Int. J. Radiat. Oncol. Biol. Phys..

[15] Tomozawa Y., Jahangiri Y., Pathak P., Kolbeck K.J., Schenning R.C., Kaufman J.A., Farsad K. (2018). Long-term toxicity after transarterial radioembolization with yttrium-90 using resin microspheres for neuroendocrine tumor liver metastases. J. Vasc. Interv. Radiol..

[16] Milano A., Gil A.V., Fabrizi E., Cremonesi M., Veronese I., Gallo S., Lanconelli N., Faccini R., Pacilio M. (2021). In Silico Validation of MCID Platform for Monte Carlo-Based Voxel Dosimetry Applied to 90Y-Radioembolization of Liver Malignancies. Appl. Sci..

[17] Pistone D., Italiano A., Auditore L., Mandaglio G., Campenní A., Baldari S., Amato E. (2022). Relevance of artefacts in99mTc-MAA SPECT scans on pre-therapy pa-tient-specific90Y TARE internal dosimetry: A GATE Monte Carlo study. Phys. Med. Biol..

[18] JCGM Joint Committee for Guides in Metrology Evaluation of Measurement Data—Guide to the Expression of Uncertainty in Measurement; JCM 100:2008. https://www.bipm.org/documents/20126/2071204/JCGM_100_2008_E.pdf.

[19] Van der Veen A.M., Cox M.G., Possolo A. (2022). GUM guidance on developing and using measurement models. Accredit. Qual. Assur..

[20] Cherry S., Sorenson J., Phelps M. (2012). Physics in Nuclear Medicine.

[21] National Electrical Manufacturers Association (2007). Performance Measurements of Gamma Cameras.

[22] Capogni M., De Felice P.A. (2014). Prototype of a portable TDCR system at ENEA. Appl. Radiat. Isot..

[23] Goedicke A., Berker Y., Verburg F., Behrendt F., Winz O., Mottaghy F. (2013). Study-Parameter Impact in Quantitative 90-Yttrium PET Imaging for Radioembolization Treatment Monitoring and Dosimetry. IEEE Trans. Med. Imaging.

[24] Gadd R., Baker M., Nijran K.S., Owens S., Thomas W., Woods M.J., Zananiri F. (2006). Measurement Good Practice Guide No. 93: Protocol for Establishing and Maintaining the Calibration of Medical Radionuclide Calibrators and Their Quality Control.

[25] Dryák P., Šolc J. (2020). Measurement of the branching ratio related to the internal pair production of Y-90. Appl. Radiat. Isot..

[26] Decay Data Evaluation Project (DDEP) Database. http://www.lnhb.fr/ddep_wg/.

[27] Fenwick A.J., Wevrett J.L., Ferreira K.M., Denis-Bacelar A.M., Robinson A.P. (2018). Quantitative imaging, dosimetry and metrology; Where do National Metrology Institutes fit in?. Appl. Radiat. Isot..

[28] Carlier T., Willowson K.P., Fourkal E., Bailey D.L., Doss M., Conti M. (2015). (90)Y -PET imaging: Exploring limitations and accuracy under conditions of low counts and high random fraction. Med. Phys..

[29] Strydhorst J., Carlier T., Dieudonné A., Conti M., Buvat I. (2016). A gate evaluation of the sources of error in quantitative 90Y-PET. Med. Phys..

[30] Capotosti A., Moretti R., Milano A., Nardini M., Cusumano D., Annunziata S., Capogni M., D’Arienzo M., Placidi L., Indovina L. (2022). Up-to-Date Optimization of the 90Y-PET/CT Reconstruction Protocol for Volumetric Quantification in Trans-Arterial Ra-dioEmbolization (TARE) Procedures in the Era of Theranostics. Appl. Sci..

[31] Tapp K.N., Lea W.B., Johnson M.S., Tann M., Fletcher J.W., Hutchins G.D. (2014). The impact of image reconstruction bias on PET/CT 90Y dosimetry after radioembolization. J. Nucl. Med. Off. Publ. Soc. Nucl. Med..

[32] Fourkal E., Veltchev I., Lin M., Koren S., Meyer J., Doss M., Yu J.Q. (2013). 3D inpatient dose reconstruction from the PET-CT imaging of 90Y microspheres for metastatic cancer to the liver: Feasibility study. Med. Phys..

[33] Sunderland J., Christian P., Kiss T. (2015). (2015) PET/CT scanner validation for clinical trials-reasons for failure, recipes for success: The Clinical Trials Network (CTN) experience. J. Nucl. Med..

[34] Park M.A., Mahmood A., Zimmerman R.E., Limpa-Amara N., Makrigiorgos G.M., Moore S.C. (2008). Adsorption of metallic radionuclides on plastic phantom walls. Med. Phys..

[35] Fenwick A., Baker M., Ferreira K., Keightley J. (2011). Comparison of Y-90 Measurements in UK Hospitals, NPL Report IR 20. https://eprintspublications.npl.co.uk/5213/1/IR20.pdf.

[36] Ferreira K., Fenwick A., Arinc A., Johansson L. (2015). Standardisation of 90Y and determination of calibration factors for 90Y microspheres (resin) for the NPL secondary ionisation chamber and a Capintec CRC-25R. Appl. Radiat. Isot..

[37] Kossert K., Bokeloh K., Ehlers M., Nähle O., Scheibe O., Schwarz U., Thieme K. (2015). Comparison of 90Y activity measurements in nuclear medicine in Germany. Appl. Radiat. Isot..

[38] Woods M., Munster A., Sephton J., Lucas S., Walsh C. (1996). Calibration of the NPL secondary standard radionuclide calibrator for 32P, 89Sr and 90Y. Nucl. Instrum. Methods Phys. Res. Sect. A Accel. Spectrometers Detect. Assoc. Equip..

[39] AAPM Task Group 181 The Selection, Use, Calibration, and Quality Assurance of Radionuclide Calibrators Used in Nuclear Medicine (2012) Report of AAPM Task Group 181. https://www.aapm.org/pubs/reports/rpt_181.pdf.

[40] Zimmerman B., Ratel G. (2005). Report of the CIPM Key Comparison CCRI(II)-K2 Y-90. Metrologia 42, 06001. https://iopscience.iop.org/article/10.1088/0026-1394/42/1A/06001/meta.

[41] Dezarn W., Kennedy A. (2007). SU-FF-T-380: Significant differences exist across institutions in 90Y activities compared to reference standard. Med. Phys..

[42] Selwyn R.G., Nicles R.J., Thomadsen B.R., DeWerd L.A., Micka J.A. (2007). A new internal pair production branching ratio of 90Y: The development of a non-destructive assay for 90Y and 90Sr. Appl. Radiat. Isot..

[43] Pibida L., Zimmerman B.E., King L., Fitzgerald R., Bergeron D.E., Napoli E., Cessna J.T. (2020). Determination of the internal pair production branching ratio of 90Y. Appl. Radiat. Isot. Incl. Data Instrum. Methods Use Agric. Ind. Med..

